# Central Dental Association of Northern New Jersey

**Published:** 1894-01

**Authors:** 


### CENTRAL DENTAL ASSOCIATION OF NORTHERN NEW JERSEY.

The regular meeting of this Association was held May 15, 1893. The President introduced the essayist of the evening, Dr. D. M. Sabater, who read his paper on "Some Diseases of the Gums: Their Cause and Treatment."

(For Dr. Sabater's paper, see page 34.)

*The President.*—Gentlemen, you have heard the paper of the evening, and it is open for discussion. I regret to say that the three who were expected to have opened the discussion are prevented from being present,—Dr. Kingsley, Dr. Eaton, and Dr. Stockton. The meeting is open to every one, members of the Society and non-members.

### DISCUSSION.

*Dr. R. Ottolengui.*—Mr. President, there is one trouble in the mouth that Dr. Sabater has not mentioned,—that is, hypertrophy of the pulp itself; and about all I have to say on that is that it is a curious fact that while in my earlier practice it was a common occurrence for me to see hypertrophied pulps, it is now a lesion rarely met with. They were usually found in molar teeth from which a large portion of the crown had been removed by caries. It could be observed as a bluish mass, and perhaps mistaken for epulis, but when removed it was found it was not this, but an hypertrophied growth of the pulp, produced by exposure, and filling the entire cavity of the tooth. I wonder why it is that I do not meet with these cases any more, while I used to see them so frequently. I have thought that perhaps it is explained by the fact that in my earlier days the practice with which I was associated was largely among a different class of people; now I operate on a class that take more care of their teeth and give more attention to hygiene. I do not know positively that that is the reason, but I wonder whether it has anything to do with it.

Now, I want to say a word about gingivitis. I have among my patients a boy who has always had gingivitis since I have known him, except when he is under treatment. It will respond nicely to treatment, and he will recover, but when he remains away for a year or a year and a half he comes back with the same trouble. The last time he came to me I observed that the gingivitis was manifested principally on one side of the mouth, and I accused the boy of chewing mostly or altogether on the other side, and he admitted it. He did a little actual cleaning with the brush, and a good deal on that side of the mouth in eating. I wanted him to follow my instructions and chew on both sides of the mouth, but I knew he would not do it; he would say yes when I told him, and go home and follow the old habit. So I gave him specific instructions to get some chewing gum and chew it for ten minutes after every meal, on both sides of the mouth. That gave him a specific operation to perform, and his mind would be centred upon chewing on that side of the mouth; and it proved as I anticipated, it really made a change in that boy's teeth. He keeps up the gum-chewing.

Another thing about that: you have noticed in the advertisements of tutti frutti that it is said to be endorsed by the learned profession. I don't know who has endorsed it, but it is so stated. It seems to me that the chewing of gum as a constant habit is deleterious, whereas the abbreviated habit, as in the case I have cited, may have some advantage. The chewing of gum for five or ten minutes immediately after a meal excites the flow of saliva at the time it is needed by the digestive apparatus, and it may be beneficial; but it is reasonable to suppose that the salivary glands will only secrete a certain quantity of saliva, and if you make a constant drain upon them by the chewing of gum, there must be an excessive flow of saliva produced, and it necessarily becomes of an inferior quality, and of very little value as an aid to digestion.

I want to relate a case of pyorrhœa which I have not yet finished. The patient reported to me within ten days with an elongated lateral incisor, twisted and loosened, and I discovered a pocket on what you might call the posterior side of the tooth. As you probably all know, I have opposed openly the implantation or replantation of teeth in diseased territory, and it is because my experience has been that bone will not build over the labial side of the teeth; for the reason that the process at that point is so thin. In this case the tooth was a great disfigurement, and I thought it might be possible to control the pyorrhœa until the tooth would become firm. I knew of no way of making the tooth go back to its original place. How that tooth became twisted is one of those things that look like a mystery, but I think the explanation is simple. The pyorrhœa loosens the tooth, and nature begins to eject it, it drops by gravitation, and as soon as this occurs it interferes with mastication, and the other teeth displace it. In all cases of protrusion of a single tooth you will find that the occlusion is at fault. The cuspid below was driving this tooth more and more to one side. I suggested to the lady, who was determined to retain the tooth, that it should be removed and replanted; and I explained the whole theory, and why I was willing to try it in this case; and that was that the pocket was at the side of the tooth where there is plenty of bone to cover it up. The tooth seemed to be attached at every part except that one. The patient consented, and I removed the tooth. I found the root covered from end to end with a deposit of serumal calculus. I could not scrape it off with a knife without using a great deal of force. I presume that it could have been removed by the trichloracetic acid which we have now. There was an attachment and deposit all around; there was no chance to make use of the scaler. The action of trichloracetic acid is not to destroy the deposit, but to destroy the attachment between the deposit and the tooth; it loosens it in a mass and it comes away. When I undertook to deepen that socket and reshape it, I found the bone carious, so that when that was removed the tooth would not stay in place; I could put it in any position I chose, but it would come away. So I said to the lady that the easiest thing to do with this tooth was to put it in the waste-basket and give it up; but this was not to me a satisfactory conclusion. I fitted a band on the tooth, and another on the adjacent tooth, put the tooth in, and took an impression in plaster, and then soldered the bands together, and cemented the lateral incisor into its band and let it get hard, then set my tooth, and cemented the other band around the central incisor. And here I want to tell you of a little trick upon which I place the utmost value, and to which more than anything else my success in that case has been due. In the first twenty-four hours after an implantation success or failure is determined. You want to avoid infection during the operation by the free use of bichloride of mercury; but would not infection after the operation be just as bad? and are we not told that the mouth is full of bacteria all the time? Would it not be a nice thing to seal up or secure the margin of the gum to the tooth? That is almost exactly what I did. Immediately before dismissing the patient, after implanting the tooth, I cover the tooth and gum thoroughly with tannin and glycerin, leaving the mouth open for five minutes until a distinct coagulum is formed. In some cases I have known that coagulum to be attached to the tooth five or six or eight days afterwards. When the patient comes back it looks like a slough, but it is the coagulated blood, and it stands there as a guard to the entrance to that cavity, and practically seals it up. I treated this tooth in that way. There was a slight inflammation and a bluish discoloration of the gum. The tooth has been replaced now for five days, and there has not been a particle of pus formed; the coagulum is still there, the gum has formed a very pretty festoon, and it looks as though it would be successful. The patient asked me one day how I would decide whether it was successful or not, and I said by not being a failure. Then she said, "How do you decide it is not a failure?" I said, "Just as soon as you see pus you may know it is a failure." If she were to come to me to-morrow with a single drop of pus visible, I would take the whole thing out and then make a plate. The tooth is held firmly in place, and bone may build down around the root.

*The President.*—I would like to say that, in connection with the paper and the remarks of Dr. Ottolengui, the question was asked by the stenographer what acid he spoke of, and another gentleman who heard the question was not familiar with it. The acid spoken of was trichloracetic acid; and I want to emphasize the remarks of Dr. Sabater and Dr. Ottolengui in regard to that remedy. I have had an experience extending a little over two years in its use, my attention being called to it by a specialist in medicine, and its effect has been wonderfully good in my hands. I find it an invaluable remedy in connection with the removal of tartar where the scaler cannot be effectively used, and I would recommend it especially to those not familiar with it. It will cleanse a tooth and root of deposits of tartar where the scaler fails to detach it. It is not painful in its application, but is rather grateful to the tissues. It is somewhat of an escharotic, but there is an absence of serum after it has been used; it gives a dry surface. I have found it invaluable in the removal of the gum when it has grown over the edge of a cavity, placing the rubber band on before the operation. Sometimes when cutting away the gum in that way there is considerable loss of blood, but the application of trichloracetic acid will enable you to remove it without hemorrhage. After the filling is completed, and examined a day or two subsequently, there will be found a healthy condition of the gum; the parts that were burned having sloughed off and healthy action taken place. I speak from personal experience with this remedy.

*Dr. Richards.*—How is the acid applied, and in what quantities? Is it by hypodermic injection or upon cotton forced into the pockets?

*Dr. Ottolengui.*—I think Dr. Van Woert's way of using it is the best, with an orange-wood stick,—making a skewer of orange wood, dipping it in the acid and putting it under the gum; in that way not carrying any more than is wanted.

Now, I want to ask a question, and I would like to have it answered. I would like to know how many gentlemen in the room have constantly in use in the office a spraying apparatus; I don't mean for spraying ether, but for spraying under the gum; how many of you have it? Practically nobody except Dr. Sabater. I bought a spraying apparatus six or seven years ago to spray ether, and I could not get along without one now. I have been a regular source of revenue to the druggists in my neighborhood by ordering spraying apparatus for all my patients. Many of my patients are now using them in the mouth for cleansing the teeth,—cleaning with a brush first and spraying afterwards. A spray will remove filth from the teeth that nothing else will. If you use a brush up and down, forward and back, something will remain that a spray will take out. And your operations in the mouth for gingivitis are not worth much without it. But if a spraying apparatus is recommended and in use every day, they will not need the constant attention of the dentist. I use a simple double-bulb atomizer. A good one is the cheapest; costs about four dollars. The principal thing that I use with it, if not the only thing, is hydronaphthol. If you have a patient with soft gums and with a generally filthy condition of the mouth, use a spray on them, and you will be amazed to see its effect. It may act like chewing gum; the patient with a spraying apparatus has a specific duty to perform, and is reminded of it and don't neglect it. Every one of you should get a spraying apparatus, and you will never be without it afterwards.

It has been suggested here that the use by patients of pumice-stone is bad. It is just as bad when the dentist makes use of it. When a corundum wheel is used the particles are forced up under the gum; this is readily removed by a spraying apparatus. I use a spray more than I do a syringe. When cleaning the teeth of tartar, some pieces will get loose and lodge under the gum; the spray effectually removes them. The same result follows the use of pumice refuse. The patients like it.

*Dr. Iredell.*—In regard to whittling orange-wood for applying acid, I save time by using toothpicks made of orange-wood. I think you can procure about a thousand of them for five cents. They are flattened on one side and pointed the other. I find them a very excellent thing to use.

*Dr. Sabater.*—In regard to the use of a spray, I began to use this about two years ago by a mere chance. I had a case brought to me by a New York dentist, a man with a broken jaw, a right lower compound fracture of the ramus. It was in the summertime, and a dental splint had been made for him. When he was brought to me I was astonished to find so fetid a breath and such a filthy condition of the mouth on the opposite side from the fracture. Dr. James E. Kelly, a surgeon of the Post-Graduate Hospital, suggested the spray, and it was used in that case. Under his direction I purchased one and used it on this man every other day; he could not come every day, he was working. In the course of nine days I used it four or five times, and I was astonished to see at the end of that time the healthy change in the mouth that was originally in such a very bad condition. I have used a spray also in some cases of necrosis, using peroxide of hydrogen as a medicament. In cleaning teeth I have used it a little, through the advice of Abbott. He said, if I would use it in every case after cleaning, and especially in bad cases, I would find better results than if I were to take all the teeth out and clean them and put them back again; and I think that is so. If we use powder, a portion of the substance is left between the teeth and in the festoons of the gum, and in twenty-four hours you will find the gum highly abraided and hypertrophied; but a spray will wash all that out. Every one of you use it and you will be glad of it.

